# The Bidirectional Association between Depressive Symptoms and Gait Speed: Evidence from the English Longitudinal Study of Ageing (ELSA)

**DOI:** 10.1371/journal.pone.0068632

**Published:** 2013-07-09

**Authors:** Panayotes Demakakos, Rachel Cooper, Mark Hamer, Cesar de Oliveira, Rebecca Hardy, Elizabeth Breeze

**Affiliations:** 1 Department of Epidemiology and Public Health, University College London, London, United Kingdom; 2 Medical Research Council Unit for Lifelong Health and Ageing, University College London, London, United Kingdom; Cardiff University, United Kingdom

## Abstract

**Background:**

Depressive symptoms and physical performance are inversely associated, but it is unclear whether their association is bidirectional. We examined whether the association between depressive symptoms and physical performance measured using gait speed is bidirectional.

**Methods:**

We used a national sample of 4,581 community-dwelling people aged 60 years and older from the English Longitudinal Study of Ageing (from 2002–03 to 2008-09). We fitted Generalized Estimating Equation (GEE) regression models to analyse repeated measurements of gait speed (m/sec) and elevated depressive symptoms (defined as a score of ≥4 on the eight-item Center for Epidemiological Studies-Depression scale).

**Results:**

Slower gait speed was associated with elevated depressive symptoms both concurrently and two years later. After adjustment for previous depressive symptoms and sociodemographic, clinical, lifestyle, psychosocial, and cognitive factors the concurrent association was partially explained (Odds Ratio [OR] 0.42, 95% confidence interval [CI], 0.30 to 0.59, per 1m/sec increase in gait speed) and the two-year lagged association fully (OR 0.75, 95% CI, 0.56 to 1.00). Elevated depressive symptoms were associated with slower gait speed. Full adjustment for covariates (including previous gait speed) partially explained both the concurrent (β regression coefficient [β] -0.038, 95% CI, -0.050 to -0.026, for participants with elevated depressive symptoms compared with those with no or one symptom) and the two-year lagged associations (β -0.017, 95% CI, -0.030 to -0.005). Subthreshold depressive symptoms (defined as a score of two or three on the eight-item Center for Epidemiological Studies-Depression scale) were also associated with slower gait speed. Full adjustment for covariates partially explained both the concurrent (β -0.029, 95% CI, -0.039 to -0.019, for participants with subthreshold symptoms compared with those with no or one symptom) and the two-year lagged associations (β -0.011, 95% CI, -0.021 to -0.001).

**Conclusions:**

The inverse association between gait speed and depressive symptoms appears to be bidirectional.

## Introduction

Evidence is accumulating that elevated depressive symptoms are a risk factor for physical disability [1–5] and poor physical performance [6–9] at older ages. Physical disability and physical performance are closely related [10] with decreasing physical performance often viewed as a precursor to physical disability [11,12]. Further, findings suggest that elevated depressive symptoms are related to the decline in physical performance of well-functioning older people [13] making depression relevant to the whole process of physical disablement [12]. In parallel, evidence has accrued that physical disability and poor physical performance predict depression and elevated depressive symptoms [3,14–18].

Despite the parallel development of these two lines of research and suggestions that depression and physical decline and disability might be mutually reinforcing [2,3,5,13,19] research on the bidirectional association between elevated depressive symptoms and physical disability [20–25] or declines in physical performance [23,26] is limited, with some studies providing evidence for a bidirectional longitudinal association [20,22,23] and other studies not [21,24–26]. This research is in addition limited by the use of samples that are selected [20,21] and not representative of the general population [20–26]. Thus, our understanding of the association between depressive symptoms and physical functioning remains poor.

We explored the bidirectional association between depressive symptoms and physical performance measured using gait speed, which is an established predictor of incident physical disability [10,27] and mortality [28–30]. We utilized repeated measurements of both gait speed and depressive symptoms over six years. We hypothesized that slow gait speed would be related to concurrent and subsequent elevation of depressive symptoms and elevated and subthreshold depressive symptoms would be related to concurrent slow gait speed and subsequent decline in gait speed. We also investigate clinical, lifestyle, psychosocial, cognitive, inflammatory, and metabolic pathways that might mediate these associations.

## Materials and Methods

### Study Population

The English Longitudinal Study of Ageing (ELSA) is a prospective observational study of community-dwelling people aged 50 years and over in England. Participants were recruited from households that had participated in the Health Survey for England in 1998, 1999, and 2001. At baseline, in 2002-03, the sample comprised 11,391 core members and was designed to be nationally representative. Follow-up interviews took place in 2004-05, 2006-07, 2008-09, and 2010-11 and health examinations in 2004-05 and 2008-09. ELSA has been approved by the National Research Ethics Service and all participants have given informed consent. Written informed consent was obtained for the health examination. The ELSA data and documentation are publicly available and can be downloaded from the UK Data Service (http://www.esds.ac.uk/findingData/snDescription.asp?sn=5050). The present work has used the 15th edition of the data.

Our study included people aged 60 years and older (n=7,246) since data on gait speed were not collected from younger participants, and used data from the first four interviews. The analytic sample comprised 4,581 individuals, after the exclusion of 1,468 individuals who were lost to follow up after the baseline and 1,197 individuals with missing baseline data in any of the variables used in the analyses. In the second part of the analysis the analytic sample comprised 4,300 individuals because of 281 additional cases with missing data.

Attrition was associated with male sex, older age, lower socioeconomic position, more comorbidities, higher rates of elevated depressive symptoms, and slower gait speed.

### Assessment of gait speed

Participants able to walk were asked to walk a distance of eight feet at their usual pace from a standing point twice. The times of the two successive walks were recorded using a stopwatch and the mean time was calculated. If a single measurement of time was available then this was used instead of the mean time. We calculated gait speed by dividing distance (in meters) by mean time (in seconds) for participants who did not use mobility aids to perform the walking test. At baseline there were 56 individuals with extreme time values (<1sec or ≥60sec) in either the first or the second walk, which were not used in the calculation of the mean time. We also derived a four-category variable of gait speed by categorizing gait speed into 0.2m/s bands with measurements of 1.0m/s or higher and lower than 0.6m/s grouped into the fastest and slowest gait speed categories, respectively, to examine whether the association between gait speed and elevated depressive symptoms was linear [29].

### Assessment of Depressive Symptoms

Depressive symptomatology was measured using the eight-item Center for Epidemiological Studies-Depression (CES-D) scale. CES-D is not a diagnostic instrument for clinical depression but can be used to identify people “at risk” of depression [31]. The psychometric values of the eight-item CES-D are comparable to those of the full 20-item CES-D [32]. Five of the eight CES-D items (i.e. felt depressed, was happy, felt lonely, enjoyed life, felt sad) were depressed mood items, while the remaining three (i.e. everything was an effort, restless sleep, and could not get going) were somatic complaints items [32]. We derived a summary CES-D score by adding responses to all eight dichotomous questions (possible range: 0-8). To identify cases of elevated depressive symptoms that are possible cases of clinical depression we dichotomized the summary score around the cut point of four or higher that corresponds to the cut point of 16 or higher on the 20-item CES-D [32]. To explore the association between subthreshold depressive symptomatology [33,34] and gait speed and thus better assess the association between depressive symptoms and physical performance, we transformed the summary CES-D score into a three-category variable with the following categories: no or one depressive symptom (CES-D score of zero or one), subthreshold depressive symptoms (CES-D score of two or three), and elevated depressive symptoms (CES-D score of four or higher).

### Covariates

Age, sex, marital status, education, total net household wealth, and self-reported doctor diagnosed cardiovascular (hypertension, angina, heart attack, congestive heart failure, heart murmur, abnormal heart rhythm, diabetes, and stroke) and non-cardiovascular (chronic lung disease, asthma, arthritis, osteoporosis, cancer, Parkinson’s disease, Alzheimer’s disease and dementia, and emotional, nervous, or psychiatric problems) comorbidities were measured and considered to be confounders. Bodily pain (no, mild, moderate, severe pain) was measured as a clinical mediator [13,24,35], while smoking (never a smoker, ex-smoker, current smoker), frequency of alcohol consumption in the last 12 months (daily, weekly/monthly, rarely/never), physical activity on a weekly basis (not at all, mild, moderate, vigorous), body mass index (weight (in kilograms)/height^2^ (in meters)) (BMI): <25, ≥25 to <30, ≥30, missing and waist circumference: <94cm, ≥94cm, ≥102cm, missing (in men) and <80cm, ≥80cm, ≥88cm, missing (in women) were measured as lifestyle mediators [2,23]. Social support (high, low, missing) from partner, children, relatives, and friends, number of problems with social relationships (none, one, two or more, missing), number of close relationships (missing/none, one to three, four to six, seven to nine, ≥10), and sense of control at home (high, moderate, low) were measured as psychosocial mediators [2,21,36–38]. Memory (score of immediate and delayed 10-word recall), and executive function (number of animals named in 60 seconds) were also assessed as covariates [2,4,13]. A time variable was also derived to indicate the temporal sequence of the four interviews (*t=1, 2, 3, 4*).

All covariates were measured at baseline and each of the follow-up interviews, except for age, sex, marital status, education, wealth, and smoking which were measured only at baseline. Data on BMI and waist circumference were not measured at baseline. They had been measured in the Health Survey for England in 1998, 1999, and 2001.

In supplementary analyses, we assessed triglycerides, high-density lipoprotein cholesterol, fibrinogen, and high sensitivity C-reactive protein from the 2004-05 health examination as mediators because of their associations with physical performance and depressive symptoms at older ages [39–41].

### Statistical analysis

#### Gait speed as a predictor of elevated depressive symptoms

After the analysis of the baseline characteristics of the sample according to gait speed categories, we used Generalized Estimating Equations (GEE) to model the association between gait speed (both continuous and categories) and elevated depressive symptoms. GEE is an extension of the generalized linear model that accounts for the within-subject correlation across repeated measurements, allows for within-subject missing data, and is appropriate to estimate population-averaged effects over time [42].

We estimated two GEE models using an unstructured correlation structure. First, we assessed the concurrent association between gait speed and elevated depressive symptoms using logistic regression. In this model gait speed at time *t* (*t*=2, 3, 4) was related to elevated depressive symptoms at time *t* with adjustment for elevated depressive symptoms at time *t-1*. The adjustment for elevated depressive symptoms at time *t-1* was made to account for the recurrent nature of depressive symptoms and compensate for our inability to adjust for depression history. Then, we estimated a 2-year lagged logistic regression model to assess the temporal dimension of this association. In this model gait speed at time *t* (*t*=1, 2, 3, 4) was related to elevated depressive symptoms at time *t+1* with adjustment for elevated depressive symptoms at time *t*. We adjusted our models initially for time, age, marital status, and sex, then for education and wealth, and then in addition for comorbidities. Mediating factors were adjusted for in the final model. In supplementary analyses (see File S1) we examined these associations in a subgroup of well-functioning (i.e. non-disabled) older people and explored the role of somatic depressive symptoms and metabolic and inflammatory pathways in this association.

#### Categories of depressive symptoms as a predictor of gait speed

We analysed the baseline characteristics of the sample by depressive symptom category (no or one symptom, subthreshold symptoms, and elevated symptoms). The modelling strategy was similar to that of the first part of the analysis. Concurrent and 2-year lagged linear regression GEE models were estimated for the association between categories of depressive symptoms and gait speed. Supplementary analyses similar to those described in the first part of the analysis were also performed (see File S1).

All *P* values were 2-sided and statistical significance was determined at *P* <.05. Statistical analyses were performed using STATA 11.

## Results

### Gait speed as a predictor of elevated depressive symptoms

The slower the gait speed the more likely the participants to be older, female, non-married, less wealthy and educated, less physically active, more often troubled by pain, obese, and more depressed. Also, the slower the gait speed the more likely the participants to have more cardiovascular and non-cardiovascular comorbidities and poorer cognitive ability; report lower sense of control at home; and consume alcohol less often (Table 1).

**Table 1:** The baseline characteristics of 4,581 participants by gait speed, English Longitudinal Study of Ageing, 2002-03.

	**Gait speed: ≥1m/s (n=1,387)**	**Gait speed: <1m/s to ≥0.8m/s (n=1,367)**	**Gait speed: <0.8m/s to ≥0.6m/s (n=1,095)**	**Gait speed: <0.6m/s (n=732)**	***Pvalue*^*a*^**
Age, mean (SD), years	67.3 (5.6)	69.5 (6.5)	71.7 (7.2)	74.0 (7.8)	*<.001*
Female, No. (%)	681 (49.1)	732 (53.6)	633 (57.8)	482 (65.9)	*<.001*
Married, No. (%)	995 (71.7)	911 (66.6)	657 (60.0)	372 (50.8)	*<.001*
University degree or equivalent, No. (%)	224 (16.2)	120 (8.8)	64 (5.8)	20 (2.7)	*<.001*
Wealthiest tertile of total net household wealth (≥£201,012), No. (%)	701 (50.5)	483 (35.3)	268 (24.5)	130 (17.8)	*<.001*
No cardiovascular disease, No. (%)	730 (52.6)	611 (44.7)	442 (40.4)	221 (30.2)	*<.001*
No non-cardiovascular disease, No. (%)	799 (57.6)	681 (49.8)	468 (42.7)	189 (25.8)	*<.001*
Never a smoker, No. (%)	529 (38.1)	486 (35.6)	384 (35.1)	260 (35.5)	*.049*
Daily or almost daily consumption of alcohol, No. (%)	498 (35.9)	393 (28.8)	282 (25.8)	154 (21.0)	*<.001*
Vigorous-intensity physical activity at least once a week, No. (%)	528 (38.1)	381 (27.9)	211 (19.3)	83 (11.3)	*<.001*
Highest quartile of memory (no. of recalled words), (12-20 words), No. (%)	467 (33.7)	360 (26.3)	180 (16.4)	82 (11.2)	*<.001*
Highest quartile of executive function (no. of named animals in 60 sec), (≥23 named animals), No. (%)	501 (36.1)	357 (26.1)	177 (16.2)	90 (12.3)	*<.001*
Not often troubled by pain, No. (%)	1,051 (75.8)	908 (66.4)	658 (60.1)	274 (37.4)	*<.001*
Highest social support from at least one source^b^, No. (%)	867 (64.4)	857 (64.8)	673 (64.8)	416 (61.6)	*.498*
No problems with social relationships^b^, No. (%)	908 (68.0)	852 (64.6)	698 (67.6)	417 (62.7)	*.078*
Ten or more close relationships, No. (%)	383 (27.6)	297 (21.7)	260 (23.7)	143 (19.5)	*<.001*
Highest sense of control at home^b^, No. (%)	807 (60.7)	743 (56.7)	523 (51.5)	280 (42.4)	*<.001*
Body Mass Index^c^ ≥30 kg/m^2^, No. (%)	233 (17.9)	317 (24.8)	272 (26.8)	200 (31.9)	*<.001*
Waist circumference^c^ ≥102cm in men and ≥88cm in women, No. (%)	371 (35.1)	451 (41.8)	395 (46.0)	319 (55.4)	*<.001*
Elevated depressive symptoms (CES-D score≥4), No. (%)	96 (6.9)	153 (11.2)	146 (13.3)	211 (28.8)	*<.001*
Gait speed, mean (SD), m/s	1.18 (0.16)	0.90 (0.58)	0.71 (0.58)	0.47 (0.11)	*<.001*

a *P* value was generated using χ^2^, analysis of variance, and Kruskal-Wallis tests, for categorical, continuous, and ordinal variables, respectively.

b Estimates of social support, number of problems with social relationships, and sense of control at home are based on 4,379, 4,352, and 4,316 individuals with non-missing data, respectively.

c Estimates of body mass index and waist circumference are based on 4,218 and 3,569 individuals with non-missing data, respectively.

The concurrent inverse associations between gait speed and gait speed categories and elevated depressive symptoms were initially significant (Table 2). Adjustment for covariates reduced the strength of these associations but did not fully attenuate them. Unlike the concurrent associations, the 2-year lagged inverse associations were explained in the fully adjusted model (Table 2). Overall, the association between gait speed and elevated depressive symptoms appeared to be linear and graded. All mediators explained relatively small amounts of these associations, except for obesity, which did not explain any (data not shown). The strongest effect was observed for psychosocial mediators (especially sense of control) and pain. Supplementary analyses indicated that both concurrent and 2-year lagged associations between gait speed and elevated depressive symptoms were significant in a subgroup of well-functioning older people (Table S1 in File S1) as well as in the absence of somatic CES-D symptoms (Table S2 in File S1) and that inflammatory and metabolic factors did not influence them (Table S3 in File S1).

**Table 2:** GEE analysis^a^ of elevated depressive symptoms by gait speed, English Longitudinal Study of Ageing, 2002-09.

	**Concurrent association^b^ (n=4,355)**	**2-year lagged association^c^ (n=4,581)**
**Predictor: gait speed (m/s)**		
**Model 1**	0.17 (0.13 to 0.22)	0.31 (0.24 to 0.40)
**Model 2**	0.19 (0.14 to 0.25)	0.36 (0.27 to 0.46)
**Model 3**	0.23 (0.17 to 0.32)	0.45 (0.34 to 0.58)
**Model 4**	0.42 (0.30 to 0.59)	0.75 (0.56 to 1.00)
**Predictor: categories of gait speed (m/s)**		
**Model 1**		
≥1	Reference category	Reference category
<1 to ≥0.8	1.31 (1.07 to 1.62)	1.07 (0.90 to 1.29)
<0.8 to ≥0.6	1.74 (1.42 to 2.14)	1.64 (1.37 to 1.96)
<0.6	3.40 (2.75 to 4.21)	2.08 (1.71 to 2.52)
*P value for linear trend*	*<.001*	*<.001*
**Model 2**		
≥1	Reference category	Reference category
<1 to ≥0.8	1.27 (1.03 to 1.57)	1.03 (0.85 to 1.23)
<0.8 to ≥0.6	1.64 (1.33 to 2.02)	1.50 (1.25 to 1.80)
<0.6	3.15 (2.53 to 3.92)	1.86 (1.52 to 2.27)
*P value for linear trend*	*<.001*	*<.001*
**Model 3**		
≥1	Reference category	Reference category
<1 to ≥0.8	1.24 (1.00 to 1.53)	0.98 (0.81 to 1.18)
<0.8 to ≥0.6	1.50 (1.21 to 1.85)	1.37 (1.14 to 1.65)
<0.6	2.73 (2.18 to 3.41)	1.57 (1.28 to 2.93)
*P value for linear trend*	*<.001*	*<.001*
**Model 4**		
≥1	Reference category	Reference category
<1 to ≥0.8	1.16 (0.93 to 1.45)	0.88 (0.73 to 1.06)
<0.8 to ≥0.6	1.27 (1.01 to 1.58)	1.13 (0.93 to 1.37)
<0.6	1.90 (1.49 to 2.43)	1.08 (0.87 to 1.35)
*P value for linear trend*	*<.001*	*<.131*

a The estimates are Odds Ratios (95% confidence intervals).

b In this part of the analysis we analysed the association between elevated depressive symptoms and gait speed measured at time *t* after adjustment for elevated depressive symptoms at time *t-1*.

c In this part of the analysis we analysed the association between elevated depressive symptoms measured at time *t+1* and gait speed measured at time *t* after adjustment for elevated depressive symptoms at time *t*.

Model 1 is adjusted for time, age, baseline marital status, sex, and elevated depressive symptoms (see above: footnotes b and c).

Model 2 is adjusted for all covariates in model 1 and baseline education and tertiles of total net household wealth.

Model 3 is adjusted for all covariates in model 2 and repeated measurements of counts of cardiovascular and non-cardiovascular comorbidities (0, 1, 2, ≥3).

Model 4 is the fully adjusted model (adjusted for all covariates in model 3 and baseline body mass index, waist circumference, and smoking, and repeated measurements of frequency of alcohol consumption, physical activity, memory, executive function, pain, social support, number of problems with social relationships, number of close relationships, and sense of control at home).

### Categories of depressive symptoms as a predictor of gait speed

The more the depressive symptoms the more likely the participants to be female, non-married, less wealthy and educated, smokers, less physically active, and more often troubled by pain; have more non-cardiovascular comorbidities and poorer executive function; report lower sense of control at home, less social support, more problems with family and friends, and fewer close social relationships; consume alcohol less often; and walk more slowly (Table 3).

**Table 3:** The baseline characteristics of 4,300 participants by depressive symptoms, English Longitudinal Study of Ageing, 2002-03.

	**CES-D score: 0-1(n=2,960)**	**CES-D score: 2-3 (n=798)**	**CES-D score: ≥4 (n=542)**	***Pvalue*^*a*^**
Age, mean (SD), years	69.4 (6.7)	70.8 (7.2)	70.3 (7.0)	*<.001*
Female, No. (%)	1,502 (50.7)	489 (61.3)	381 (70.3)	*<.001*
Married, No. (%)	2,073 (70.0)	473 (59.3)	248 (45.8)	*<.001*
University degree or equivalent, No. (%)	332 (11.2)	50 (6.3)	23 (4.2)	*<.001*
Wealthiest tertile of total net household wealth (≥£201,012), No. (%)	1,185 (40.0)	231 (29.0)	105 (19.4)	*<.001*
No cardiovascular disease, No. (%)	1,400 (47.3)	305 (38.2)	210 (38.8)	*<.001*
No non-cardiovascular disease, No. (%)	1,601 (54.1)	280 (35.1)	171 (31.6)	*<.001*
Never a smoker, No. (%)	1,090 (36.8)	283 (35.5)	185 (34.1)	*<.001*
Daily or almost daily consumption of alcohol, No. (%)	909 (30.7)	219 (27.4)	125 (23.1)	*<.001*
Vigorous-intensity physical activity at least once a week, No. (%)	895 (30.2)	175 (21.9)	90 (16.6)	*<.001*
Highest quartile of memory (no. of recalled words), (12-20 words), No. (%)	808 (27.3)	149 (18.7)	105 (19.4)	*<.001*
Highest quartile of executive function (no. of named animals in 60 sec), (≥23 named animals), No. (%)	832 (28.1)	178 (22.3)	84 (15.5)	*<.001*
Not often troubled by pain, No. (%)	2,129 (71.9)	397 (49.8)	236 (43.5)	*<.001*
Highest social support from at least one source^b^, No. (%)	1,916 (66.9)	449 (59.6)	270 (53.6)	*<.001*
No problems with social relationships^b^, No. (%)	1,990 (69.8)	452 (60.5)	270 (54.2)	*<.001*
Ten or more close relationships, No. (%)	756 (25.5)	173 (21.7)	91 (16.8)	*<.001*
Highest sense of control at home^b^, No. (%)	1,708 (60.2)	355 (48.0)	181 (36.9)	*<.001*
Body Mass Index^c^ ≥30 kg/m^2^, No. (%)	617 (22.4)	185 (25.0)	143 (29.6)	*.008*
Waist circumference^c^ ≥102cm in men and ≥88cm in women, No. (%)	927 (39.9)	296 (47.6)	197 (47.2)	*.001*
Gait speed, mean (SD), m/s	0.93 (0.25)	0.80 (0.26)	0.76 (0.27)	*<.001*

a *P* value was generated using χ^2^, analysis of variance, and Kruskal-Wallis tests, for categorical, continuous, and ordinal variables, respectively.

b Estimates of social support, number of problems with social relationships, and sense of control at home are based on 4,123, 4,098, and 4,066 individuals with non-missing data, respectively.

c Estimates of body mass index and waist circumference are based on 3,979 and 3,361 individuals with non-missing data, respectively.

Both concurrent and 2-year lagged associations between categories of depressive symptoms and gait speed were significant and remained so in the fully adjusted model (Table 4). These findings reveal the existence of a gradient; the more the depressive symptoms of an older person, the slower their gait speed. Health behaviours, pain, and psychosocial factors partially explained these associations (data not shown). The exclusion of disabled participants (Table S4 in File S1), the absence of somatic CES-D symptoms (Table S5 in File S1), and the adjustment for inflammatory and metabolic factors did not influence much these associations (Table S6 in File S1).

**Table 4:** GEE analysis^a^ of gait speed by depressive symptoms, English Longitudinal Study of Ageing, 2002-09.

	**Concurrent association^b^ (n=4,285)**	**2-year lagged association^c^ (n=4,300)**
**Model 1**		
No or one symptom (CES-D score: 0-1)	1 [reference]	1 [reference]
Subthreshold symptoms (CES-D score: 2-3)	-0.053 (-0.063 to -0.043)	-0.028 (-0.038 to -0.018)
Elevated symptoms (CES-D score: ≥4)	-0.073 (-0.085 to -0.062)	-0.048 (-0.060 to -0.036)
*P value for linear trend*	*<.001*	*<.001*
**Model 2**		
No or one symptom (CES-D score: 0-1)	1 [reference]	1 [reference]
Subthreshold symptoms (CES-D score: 2-3)	-0.050 (-0.060 to -0.040)	-0.026 (-0.036 to -0.016)
Elevated symptoms (CES-D score: ≥4)	-0.071 (-0.082 to -0.059)	-0.042 (-0.054 to -0.030)
*P value for linear trend*	*<.001*	*<.001*
**Model 3**		
No or one symptom (CES-D score: 0-1)	1 [reference]	1 [reference]
Subthreshold symptoms (CES-D score: 2-3)	-0.045 (-0.055 to -0.035)	-0.022 (-0.032 to -0.012)
Elevated symptoms (CES-D score: ≥4)	-0.064 (-0.076 to -0.052)	-0.036 (-0.048 to -0.024)
*P value for linear trend*	*<.001*	*<.001*
**Model 4**		
No or one symptom (CES-D score: 0-1)	1 [reference]	1 [reference]
Subthreshold symptoms (CES-D score: 2-3)	-0.029 (-0.039 to -0.019)	-0.011 (-0.021 to -0.001)
Elevated symptoms (CES-D score: ≥4)	-0.038 (-0.050 to -0.026)	-0.017 (-0.030 to -0.005)
*P value for linear trend*	*<.001*	*.002*

a The estimates are β regression coefficients (95% confidence intervals).

b In this part of the analysis we analysed the association between gait speed and categories of depressive symptoms measured at time *t* after adjustment for gait speed at time *t-1*.

c In this part of the analysis we analysed the association between gait speed measured at time *t+1* and CES-D score categories measured at time *t* after adjustment for gait speed at time *t*.

Model 1 is adjusted for time, age, baseline marital status, sex, and gait speed (see above: footnotes b and c).

Model 2 is adjusted for all covariates in model 1 and baseline education and tertiles of total net household wealth.

Model 3 is adjusted for all covariates in model 2 and repeated measurements of the count of cardiovascular and non-cardiovascular comorbidities (0, 1, 2, ≥3).

Model 4 is the fully adjusted model (adjusted for all covariates in model 3 and baseline body mass index, waist circumference, and smoking, and repeated measurements of frequency of alcohol consumption, physical activity, memory, executive function, pain, social support, number of problems with social relationships, number of close relationships, and sense of control at home).

## Discussion

In a national sample of people aged 60 years and over, we found the association between gait speed and depressive symptoms to be bidirectional. People with slower gait speed are currently and in the future more likely to report elevated depressive symptoms. Further, people who reported subthreshold or elevated depressive symptoms have slower gait speed and are likely to experience a decrease in their gait speed and thus physical performance in the future. Adjustment for sociodemographic, clinical, lifestyle, psychosocial, cognitive, inflammatory, and metabolic factors only partially explained these associations, except for the association between gait speed and later elevated depressive symptoms, which was fully explained. These associations were not an artefact of the overlap between physical performance and somatic symptoms of depression and appear to be relevant to the entire process of disablement as they remained robust in a subgroup of well-functioning older people.

To our knowledge this is the first study to examine the bidirectional association between gait speed and depressive symptomatology and its temporal dimension using multiple time points and a national sample. An earlier study in Korea found a bidirectional association [23], but this study was limited by the use of a smaller regional sample and only two time points over one-year follow-up. A recent study from the Netherlands that used data from the Longitudinal Aging Study Amsterdam (LASA) did not find a bidirectional association, but they used a different design and thus their findings are not directly comparable to ours [26].

### Gait speed as a predictor of elevated depressive symptoms

Our findings that gait speed is inversely associated with both concurrent and future elevated depressive symptoms independent of earlier elevated depressive symptoms are new and need to be confirmed by future research. They concur with the findings of most [15,21,22,43] but not all [25] previous studies that explored the association between physical disability and depressive symptoms using multiple time points, despite inconsistencies about whether physical disability is associated with subsequent depressive symptoms [22] or not [21,43].

The strong association between slower gait speed and elevated depressive symptoms points to the need to screen for depression among older adults with decreased physical performance. The attenuation of the 2-year lagged association between slower gait speed and future elevated depressive symptoms after full adjustment suggests that the process of elevation of depressive symptoms in older people with slower gait speed is likely complex and multifactorial involving clinical, lifestyle, cognitive, and psychosocial pathways. The persistence of the concurrent association probably indicates the existence of pathways unaccounted for in our study.

### Categories of depressive symptoms as a predictor of gait speed

We found that older people with elevated depressive symptoms are more likely to experience, both concurrently and in the future, slower gait speed independent of their gait speed earlier. Evidence on this is scarce with just one study showing a significant concurrent association among women in mid-life [35] and no study on the lagged association. Findings on the association between repeated measurements of depressive symptoms and physical disability largely concur with ours [20,22,25,34,44] with only one study finding a lagged association but failing to find a concurrent association [21]. These findings point to depression as a modifiable risk that needs to be targeted by disability prevention programs at older ages.

We also found that older people with subthreshold depressive symptoms are more likely to experience slower gait speed compared with their counterparts with no or one depressive symptom. These findings highlight the existence of two or more depressive symptoms as a threshold to identify those at greater risk of physical decline either concurrently or two years later. Previous studies have found baseline subthreshold depressive symptoms to be related to physical performance [9] and disability [45], but these studies did not use repeated measurements of depressive symptomatology. To our knowledge, only one study has explored the lagged, but not the concurrent, association between subthreshold symptomatology and physical disability using repeated measurements [34]. They found a significant association between the subthreshold depressive symptoms and subsequent physical disability and that subthreshold depressive symptomatology served as the threshold over which additional symptoms did not add to the risk of physical disability. We found that the strength of the association between subthreshold depressive symptoms and gait speed was comparable to that of the association between elevated depressive symptoms and gait speed. Future research needs to explore this issue further. These findings could also inform disability prevention programs at older ages.

### Limitations & Strengths

The investigation of the bidirectional association between an objective measure of physical performance i.e. gait speed and depressive symptoms and the exploration of both concurrent and 2-year lagged associations over a period of 6 years, as well as the use of repeated measurements of outcomes, predictors, and covariates are strengths of our work. The use of a national sample makes our results altogether more generalizable to the general community-dwelling population of people aged 60 years or older.

Selective attrition is a concern for our study as it is in most ageing cohorts. We hypothesize that our results represent a conservative estimate of the association between depressive symptoms and physical performance, given that attrition is associated with both elevated depressive symptoms and slower gait speed. Future research needs to replicate our work by exploring the trajectories of depressive symptoms and gait speed over longer periods of follow-up and the potentially bidirectional associations between other measures of physical performance i.e. balance or upper extremity function and depressive symptoms. Further, studies are required to explore the causal pathways and ascertain whether interventions to improve physical performance can reduce depression and whether interventions to reduce depression can improve physical performance at older ages [46].

Our work supports claims that the elevation of depressive symptoms and declines in physical performance are mutually related at older ages. Our main findings indicate that physical disability and depressive symptoms should be treated as comorbid in older people. These findings may prove useful both for prevention strategies and screening in clinical practice.

## Supporting Information

File S1 Table S1. GEE analysis of elevated depressive symptoms by gait speed, English Longitudinal Study of Ageing, 2002-2009. These models were based on well-functioning older people who reported no limitations in Activities of Daily Living/Instrumental Activities of Daily Living at baseline. Table S2. GEE analysis of different versions of the CES-D score by gait speed, English Longitudinal Study of Ageing, 2002-2009. Table S3. GEE analysis of elevated depressive symptoms by gait speed, English Longitudinal Study of Ageing, 2004-2009. These models were in addition adjusted for inflammatory and metabolic indicators. Table S4. GEE analysis of gait speed by depressive symptoms, English Longitudinal Study of Ageing, 2002-2009. These models were based on well-functioning older people who reported no limitations in Activities of Daily Living/Instrumental Activities of Daily Living at baseline. Table S5. GEE analysis of gait speed by different versions of the CES-D score, English Longitudinal Study of Ageing, 2002-2009. Table S6. GEE analysis of gait speed by depressive symptoms, English Longitudinal Study of Ageing, 2004-2009. These models were in addition adjusted for inflammatory and metabolic indicators.
(DOC)
