# Supplementary material for: The Bidirectional Association between Depressive Symptoms and Gait Speed: Evidence from the English Longitudinal Study of Ageing (ELSA)
Source: PLoS One. 2013 Jul 9;8(7):e68632. doi: 10.1371/journal.pone.0068632 (PMC3706406; doi:10.1371/journal.pone.0068632)
Supplement: File S1 — Table S1. GEE analysis of elevated depressive symptoms by gait speed, English Longitudinal Study of Ageing, 2002-2009. These models were based on well-functioning older people who reported no limitations in Activities of Daily Living/Instrumental Activities of Daily Living at baseline. Table S2. GEE analysis of different versions of the CES-D score by gait speed, English Longitudinal Study of Ageing, 2002-2009. Table S3. GEE analysis of elevated depressive symptoms by gait speed, English Longitudinal Study of Ageing, 2004-2009. These models were in addition adjusted for inflammatory and metabolic indicators. Table S4. GEE analysis of gait speed by depressive symptoms, English Longitudinal Study of Ageing, 2002-2009. These models were based on well-functioning older people who reported no limitations in Activities of Daily Living/Instrumental Activities of Daily Living at baseline. Table S5. GEE analysis of gait speed by different versions of the CES-D score, English Longitudinal Study of Ageing, 2002-2009. Table S6. GEE analysis of gait speed by depressive symptoms, English Longitudinal Study of Ageing, 2004-2009. These models were in addition adjusted for inflammatory and metabolic indicators. (DOC) [file pone.0068632.s001.doc]

**Supporting Information: Supplementary analyses**

**List of Supporting Tables**

**Gait speed as a predictor of elevated depressive symptoms**

**Table S1** presents the GEE analysis of elevated depressive symptoms (CES-D score≥4) by gait speed in a sample of well-functioning older people who reported no limitations in Activities of Daily Living/Instrumental Activities of Daily Living at baseline.

**Table S2** presents the GEE analysis of two different versions of the continuous CES-D score (i.e. the eight- and the five-item versions) by gait speed. The five-item CES-D score did not include the three somatic symptoms items that the eight-item CES-D score included i.e. “Much of the time during the past week you felt that everything you did was an effort?”, “Much of the time during the past week your sleep was restless?” and “Much of the time during the past week you could not get going?”.

**Table S3** presents the GEE analysis of elevated depressive symptoms (CES-D score≥4) by gait speed. All four models were adjusted for all covariates used in the main analyses. In addition, model A was adjusted for lipids (high-density lipoprotein cholesterol and triglycerides) and model C for inflammatory markers (fibrinogen and high sensitivity C-reactive protein). Models B and D were not adjusted for lipids and inflammatory markers, respectively, and were estimated for comparison purposes.

**Categories of depressive symptoms as a predictor of gait speed**

**Table S4** presents the GEE analyses of gait speed by depressive symptom category in a sample of well-functioning older people who reported no limitations in Activities of Daily Living/Instrumental Activities of Daily Living at baseline.

**Table S5** presents the GEE analyses of gait speed by two different versions of the CES-D score (i.e. the eight- and the five-item versions). The five-item CES-D score did not include the three somatic symptoms items that the eight-item CES-D score included i.e. “Much of the time during the past week you felt that everything you did was an effort?”, “Much of the time during the past week your sleep was restless?” and “Much of the time during the past week you could not get going?”.

**Table S6** presents the GEE analyses of gait speed by depressive symptom category. All four models were adjusted for all covariates used in the main analyses. In addition, model A was adjusted for lipids (high-density lipoprotein cholesterol and triglycerides) and model C for inflammatory markers (fibrinogen and high sensitivity C-reactive protein). Models B and D were not adjusted for lipids and inflammatory markers, respectively, and were estimated for comparison purposes.

| **Table S1. GEE analysis^a^ of elevated depressive symptoms by gait speed, English Longitudinal Study of Ageing, 2002-2009** | | |
| --- | --- | --- |
|  | **Concurrent association^b^ (n=3,265)** | **2–year lagged association^c^ (n=3,362)** |
| **Model 1** | 0.25 (0.17 to 0.36) | 0.53 (0.37 to 0.74) |
| **Model 2** | 0.28 (0.19 to 0.40) | 0.62 (0.43 to 0.87) |
| **Model 3** | 0.32 (0.22 to 0.47) | 0.70 (0.49 to 0.99) |
| **Model 4** | 0.51 (0.34 to 0.76) | 0.94 (0.65 to 1.36) |
| ^a^The estimates are Odds Ratios (95% confidence intervals). All models are estimated only for people who did not report any limitations in Activities of Daily Living and Instrumental Activities of Daily Living at baseline.  ^b^In this part of the analysis we analyzed the association between elevated depressive symptoms and gait speed at time *t* after adjustment for elevated depressive symptoms at time *t-1*.  ^c^In this part of the analysis we analyzed the association between elevated depressive symptoms at time *t+*1and gait speed at time *t* after adjustment for elevated depressive symptoms at time *t*.  Model 1 is adjusted for time, age, baseline marital status, sex, and elevated depressive symptoms (see above: footnotes b and c).  Model 2 is adjusted for all covariates in model 1 and baseline education and tertiles of total net household wealth.  Model 3 is adjusted for all covariates in model 2 and repeated measurements of the counts of cardiovascular and non-cardiovascular comorbidities (0, 1, 2, ≥3).  Model 4 is the fully adjusted model (adjusted for all covariates in model 3 and baseline body mass index, waist circumference, and smoking, and repeated measurements of frequency of alcohol consumption, physical activity, memory, executive function, pain, social support, number of problems with social relationships, number of close relationships, and sense of control at home). | | |

| **Table S2. GEE analysis^a^ of different versions of the CES-D score by gait speed, English Longitudinal Study of Ageing, 2002-2009** | | | | |
| --- | --- | --- | --- | --- |
|  | **Concurrent association^b^ (n=4,355)** | | **2-year lagged association^c^ (n=4,581)** | |
|  | **Outcome measure: the eight-item CES-D score** | **Outcome measure: the five-item CES-D score^d^** | **Outcome measure: the eight-item CES-D score** | **Outcome measure: the five-item CES-D score^d^** |
| **Model 1** | -0.81 (-0.93 to -0.70) | -0.42 (-0.50 to -0.34) | -0.49 (-0.60 to -0.37) | -0.26 (-0.33 to -0.18) |
| **Model 2** | -0.78 (-0.90 to -0.67) | -0.41 (-0.49 to -0.32) | -0.43 (-0.54 to -0.31) | -0.23 (-0.31 to -0.15) |
| **Model 3** | -0.70 (-0.82 to -0.58) | -0.36 (-0.44 to -0.28) | -0.33 (-0.45 to -0.22) | -0.17 (-0.26 to -0.09) |
| **Model 4** | -0.43 (-0.56 to -0.30) | -0.22 (-0.31 to -0.13) | -0.12 (-0.25 to 0.00) | -0.03 (-0.12 to 0.05) |
| ^a^The estimates are β regression coefficients (95% CI).  ^b^In this part of the analysis we analyzed the association between CES-D score and gait speed at time *t* after adjustment for CES-D score at time *t-1*.  ^c^In this part of the analysis we analyzed the association between CES-D score at time *t+*1and gait speed at time *t* after adjustment for CES-D score at time *t*.  ^d^The five-item CES-D score does not include the three somatic symptoms of the eight-item CES-D (i.e. “Much of the time during the past week you felt that everything you did was an effort?”, “Much of the time during the past week your sleep was restless?” and “Much of the time during the past week you could not get going?”) (score range:0-5).  Model 1 is adjusted for time, age, baseline marital status, sex, and CES-D score (see above: footnotes b and c).  Model 2 is adjusted for all covariates in model 1 and baseline education and tertiles of total net household wealth.  Model 3 is adjusted for all covariates in model 2 and repeated measurements of the counts of cardiovascular and non-cardiovascular comorbidities (0, 1, 2, ≥3).  Model 4 is the fully adjusted model (adjusted for all covariates in model 3 and baseline body mass index, waist circumference, and smoking, and repeated measurements of frequency of alcohol consumption, physical activity, memory, executive function, pain, social support, number of problems with social relationships, number of close relationships, and sense of control at home). | | | | |

| **Table S3. GEE analysis^a^ of elevated depressive symptoms by gait speed, English Longitudinal Study of Ageing, 2004-2009** | | |
| --- | --- | --- |
|  | **Concurrent association^b^ (n=2,947)** | **2–year lagged association^c^ (n=3,052)** |
| **Model A** | 0.45 (0.30 to 0.67) | 0.80 (0.56 to 1.15) |
| **Model B** | 0.45 (0.30 to 0.67) | 0.80 (0.56 to 1.15) |
|  | **Concurrent association^b^ (n=2,894)** | **2–year lagged association^c^ (n=2,997)** |
| **Model C** | 0.43 (0.29 to 0.65) | 0.78 (0.54 to 1.13) |
| **Model D** | 0.43 (0.28 to 0.65) | 0.77 (0.54 to 1.11) |
| ^a^The estimates are Odds Ratios (95% confidence intervals).  ^b^In this part of the analysis we analyzed the association between elevated depressive symptoms and gait speed at time *t* after adjustment for elevated depressive symptoms at time *t-1*.  ^c^In this part of the analysis we analyzed the association between elevated depressive symptoms at time *t+1*and gait speed at time *t* after adjustment for elevated depressive symptoms at time *t*.  All models are adjusted for time, elevated depressive symptoms (see above: footnotes b and c), the following baseline characteristics: age, sex, marital status, education, tertiles of total net household wealth, body mass index, waist circumference, and smoking, and repeated measurements of the following characteristics: counts of cardiovascular and non-cardiovascular comorbidities (0, 1, 2, ≥3), frequency of alcohol consumption, physical activity, memory, executive function, pain, social support, number of problems with social relationships, number of close relationships, and sense of control at home.  Model A is in addition adjusted for high-density lipoprotein cholesterol (mmol/L) and triglycerides (mmol/L) in 2004-05.  Model B is estimated for the same sample as Model A, but is not adjusted for high-density lipoprotein-cholesterol (mmol/L) and triglycerides (mmol/L).  Model C is in addition adjusted for fibrinogen (g/L) and high sensitivity C-reactive protein (mg/L) in 2004-05.  Model D is estimated for the same sample as Model C, but is not adjusted for fibrinogen (g/L) and high sensitivity C-reactive protein (mg/L). | | |

| **Table S4. GEE analysis^a^ of gait speed by depressive symptoms, English Longitudinal Study of Ageing, 2002-2009** | | |
| --- | --- | --- |
|  | **Concurrent association^b^ (n=3,226)** | **2-year lagged association^c^ (n=3,232)** |
| **Model 1** |  |  |
| No or one symptom (CES-D score: 0-1) | 1 [reference] | 1 [reference] |
| Subthreshold symptoms (CES-D score: 2-3) | -0.046 (-0.058 to -0.034) | -0.021 (-0.033 to -0.009) |
| Elevated symptoms (CES-D score: ≥4) | -0.063 (-0.079 to -0.048) | -0.039 (-0.054 to -0.023) |
| *P value for linear trend* | *<.001* | *<.001* |
| **Model 2** |  |  |
| No or one symptom (CES-D score: 0-1) | 1 [reference] | 1 [reference] |
| Subthreshold symptoms (CES-D score: 2-3) | -0.043 (-0.055 to -0.031) | -0.019 (-0.031 to -0.006) |
| Elevated symptoms (CES-D score: ≥4) | -0.060 (-0.075 to -0.046) | -0.033 (-0.048 to -0.017) |
| *P value for linear trend* | *<.001* | *<.001* |
| **Model 3** |  |  |
| No or one symptom (CES-D score: 0-1) | 1 [reference] | 1 [reference] |
| Subthreshold symptoms (CES-D score: 2-3) | -0.040 (-0.052 to -0.028) | -0.016 (-0.028 to -0.004) |
| Elevated symptoms (CES-D score: ≥4) | -0.056 (-0.071 to -0.041) | -0.030 (-0.046 to -0.014) |
| *P value for linear trend* | *<.001* | *<.001* |
| **Model 4** |  |  |
| No or one symptom (CES-D score: 0-1) | 1 [reference] | 1 [reference] |
| Subthreshold symptoms (CES-D score: 2-3) | -0.025 (-0.037 to -0.013) | -0.008 (-0.020 to 0.005) |
| Elevated symptoms (CES-D score: ≥4) | -0.033 (-0.049 to -0.018) | -0.013 (-0.029 to 0.003) |
| *P value for linear trend* | *<.001* | *.065* |
| ^a^The estimates are β regression coefficients (95% confidence intervals). All models are estimated only for people who did not report any limitations in Activities of Daily Living and Instrumental Activities of Daily Living at baseline.  ^b^In this part of the analysis we analyzed the association between gait speed and CES-D score categories at time *t* after adjustment for gait speed at time *t-1*.  ^c^In this part of the analysis we analyzed the association between gait speed at time *t+1* and CES-D score categories at time *t* after adjustment for gait speed at time *t*.  Model 1 is adjusted for time, age, baseline marital status, sex, and gait speed (see above: footnotes b and c).  Model 2 is adjusted for all covariates in model 1 and baseline education and tertiles of total net household wealth.  Model 3 is adjusted for all covariates in model 2 and repeated measurements of the counts of cardiovascular and non-cardiovascular comorbidities (0, 1, 2, ≥3).  Model 4 is the fully adjusted model (adjusted for all covariates in model 3 and baseline body mass index, waist circumference, and smoking, and repeated measurements of frequency of alcohol consumption, physical activity, memory, executive function, pain, social support, number of problems with social relationships, number of close relationships, and sense of control at home). | | |

| **Table S5. GEE analysis^a^ of gait speed by different versions of the CES-D score, English Longitudinal Study of Ageing, 2002-2009** | | |
| --- | --- | --- |
|  | **Concurrent association^b^ (n=4,285)** | **2-year lagged association^c^ (n=4,300)** |
| **Predictor: the eight-item CES-D score** |  |  |
| Model 1 | -0.016 (-0.019 to -0.014) | -0.010 (-0.012 to -0.008) |
| Model 2 | -0.016 (-0.018 to -0.014) | -0.009 (-0.011 to -0.007) |
| Model 3 | -0.014 (-0.017 to -0.012) | -0.008 (-0.010 to -0.005) |
| Model 4 | -0.009 (-0.011 to -0.007) | -0.004 (-0.006 to -0.001) |
| **Predictor: the five-item CES-D score^d^** |  |  |
| Model 1 | -0.018 (-0.021 to -0.014) | -0.010 (-0.013 to -0.007) |
| Model 2 | -0.017 (-0.020 to -0.014) | -0.009 (-0.012 to -0.005) |
| Model 3 | -0.015 (-0.019 to -0.012) | -0.007 (-0.010 to -0.004) |
| Model 4 | -0.009 (-0.013 to -0.006) | -0.002 (-0.006 to 0.001) |
| ^a^The estimates are β regression coefficients (95% confidence intervals).  ^b^In this part of the analysis we analyzed the association between gait speed and CES-D score at time *t* after adjustment for gait speed at time *t-1*.  ^c^In this part of the analysis we analyzed the association between gait speed at time *t+1* and CES-D score at time *t* after adjustment for gait speed at time *t*.  ^d^The five-item CES-D score did not include the three somatic symptoms of the eight-item CES-D score (i.e. “Much of the time during the past week you felt that everything you did was an effort?”, “Much of the time during the past week your sleep was restless?” and “Much of the time during the past week you could not get going?”) (score range: 0-5).  Model 1 is adjusted for time, age, baseline marital status, sex, and gait speed (see above: footnotes b and c).  Model 2 is adjusted for all covariates in model 1 and baseline education and tertiles of total net household wealth.  Model 3 is adjusted for all covariates in model 2 and repeated measurements of the counts of cardiovascular and non-cardiovascular comorbidities (0, 1, 2, ≥3).  Model 4 is the fully adjusted model (adjusted for all covariates in model 3 and baseline body mass index, waist circumference, and smoking, and repeated measurements of frequency of alcohol consumption, physical activity, memory, executive function, pain, social support, number of problems with social relationships, number of close relationships, and sense of control at home). | | |

| **Table S6. GEE analysis^a^ of gait speed by depressive symptoms, English Longitudinal Study of Ageing, 2004-2009** | | |
| --- | --- | --- |
|  | **Concurrent association^b^ (n=2,913)** | **2-year lagged association^c^ (n=2,918)** |
| **Model A^d^** |  |  |
| No or one symptom (CES-D score: 0-1) | 1 [reference] | 1 [reference] |
| Subthreshold symptoms (CES-D score: 2-3) | -0.031 (-0.043 to -0.019) | -0.004 (-0.016 to 0.008) |
| Elevated symptoms (CES-D score: ≥4) | -0.039 (-0.054 to -0.024) | -0.016 (-0.031 to -0.003) |
| *P value for linear trend* | *<.001* | *0.057* |
| **Model B** |  |  |
| No or one symptom (CES-D score: 0-1) | 1 [reference] | 1 [reference] |
| Subthreshold symptoms (CES-D score: 2-3) | -0.031 (-0.043 to -0.019) | -0.004 (-0.016 to 0.008) |
| Elevated symptoms (CES-D score: ≥4) | -0.039 (-0.054 to -0.024) | -0.016 (-0.031 to -0.003) |
| *P value for linear trend* | *<.001* | *0.057* |
|  | **Concurrent association^b^ (n=2,858)** | **2-year lagged association^c^ (n=2,863)** |
| **Model C** |  |  |
| No or one symptom (CES-D score: 0-1) | 1 [reference] | 1 [reference] |
| Subthreshold symptoms (CES-D score: 2-3) | -0.031 (-0.043 to -0.019) | -0.003 (-0.015 to 0.009) |
| Elevated symptoms (CES-D score: ≥4) | -0.039 (-0.054 to -0.024) | -0.016 (-0.031 to -0.001) |
| *P value for linear trend* | *<.001* | *0.068* |
| **Model D** |  |  |
| No or one symptom (CES-D score: 0-1) | 1 [reference] | 1 [reference] |
| Subthreshold symptoms (CES-D score: 2-3) | -0.031 (-0.043 to -0.019) | -0.003 (-0.015 to 0.009) |
| Elevated symptoms (CES-D score: ≥4) | -0.039 (-0.054 to -0.024) | -0.016 (-0.031 to -0.001) |
| *P value for linear trend* | *<.001* | *0.067* |
| ^a^The estimates are β regression coefficients (95% confidence intervals).  ^b^In this part of the analysis we analyzed the association between gait speed and CES-D score categories at time *t* after adjustment for gait speed at time *t-1*.  ^c^In this part of the analysis we analyzed the association between gait speed at time *t+1* and CES-D score categories at time *t* after adjustment for gait speed at time *t*.  ^d^All models are adjusted for time, gait speed (see above: footnotes b and c), the following baseline characteristics: age, sex, marital status, education, tertiles of total net household wealth, body mass index, waist circumference, and smoking, and repeated measurements of the following characteristics: counts of cardiovascular and non-cardiovascular comorbidities (0, 1, 2, ≥3), frequency of alcohol consumption, physical activity, memory, executive function, pain, social support, number of problems with social relationships, number of close relationships, and sense of control at home.  Model A was in addition adjusted for high-density lipoprotein cholesterol (mmol/L) and triglycerides (mmol/L) in 2004-05.  Model B was estimated for the same sample as model A, but was not adjusted for high-density lipoprotein-cholesterol (mmol/L) and triglycerides (mmol/L).  Model C was in addition adjusted for fibrinogen (g/L) and high sensitivity C-reactive protein (mg/L) in 2004-05.  Model D was estimated for the same sample as model C, but was not adjusted for fibrinogen (g/L) and high sensitivity C-reactive protein (mg/L). | | |
